# Patterns of Expression in the Matrix Proteins Responsible for Nucleation and Growth of Aragonite Crystals in Flat Pearls of *Pinctada fucata*

**DOI:** 10.1371/journal.pone.0066564

**Published:** 2013-06-12

**Authors:** Liang Xiang, Jingtan Su, Guilan Zheng, Jian Liang, Guiyou Zhang, Hongzhong Wang, Liping Xie, Rongqing Zhang

**Affiliations:** 1 Institute of Marine Biotechnology, School of Life Sciences, Tsinghua University, Beijing, China; 2 Protein Science Laboratory of the Ministry of Education, Tsinghua University, Beijing, China; National Institutes of Health, United States of America

## Abstract

The initial growth of the nacreous layer is crucial for comprehending the formation of nacreous aragonite. A flat pearl method in the presence of the inner-shell film was conducted to evaluate the role of matrix proteins in the initial stages of nacre biomineralization *in vivo*. We examined the crystals deposited on a substrate and the expression patterns of the matrix proteins in the mantle facing the substrate. In this study, the aragonite crystals nucleated on the surface at 5 days in the inner-shell film system. In the film-free system, the calcite crystals nucleated at 5 days, a new organic film covered the calcite, and the aragonite nucleated at 10 days. This meant that the nacre lamellae appeared in the inner-shell film system 5 days earlier than that in the film-free system, timing that was consistent with the maximum level of matrix proteins during the first 20 days. In addition, matrix proteins (Nacrein, MSI60, N19, N16 and Pif80) had similar expression patterns in controlling the sequential morphologies of the nacre growth in the inner-film system, while these proteins in the film-free system also had similar patterns of expression. These results suggest that matrix proteins regulate aragonite nucleation and growth with the inner-shell film *in vivo*.

## Introduction

Nacre is the lustrous internal ‘mother of pearl’ layer of many molluscan shells, and pearls themselves are structurally similar to the nacre of pearl oyster shells [1]. The structure of nacre is a brick and mortar arrangement: the bricks are flat polygonal crystals of aragonite, and the mortar is made out of polysaccharide chitin, lipids and proteins laid down orthogonally to each other and aligned with the aragonite crystal axes. This structure result in higher mechanical strength and osteoinductive activity compared with inorganic CaCO_3_ crystals [2]–[3]. The accurate and orderly assembly of the crystals and organic matrix imparts lustre by reflecting light and causing uniform interference from the layered compartment structure. The shell of the pearl oyster, *Pinctada fucata*, typically consists of an outermost organic layer called the periostracum and calcium carbonate oriented in two distinct structures: the outer calcite prismatic layer and the inner aragonite nacreous layer [4]. The nacre from the shell has become one of the most intensively studied biological structures in the fields of biology, physics, chemistry and materials science. Understanding the process by which living organisms control the growth of structured inorganic materials could lead to significant advances in materials science, and open the door to novel synthesis techniques for nanoscale composites [5]–[7].

Although aragonite crystals may be attained by using an organic template or heteroepitaxy, the molecular aspects of nacre building are still far from being fully understood. In molluscs, nacre formation occurs in the extrapallial fluid, located in an extracellular cavity between the mantle and shell [8]. The mantle was thought to control the nacre growth in the pearl-forming oyster *Pinctada fucata*. The epithelial cells from mantle tissue secrete organic components such as proteins and polysaccharides, playing an important role in the spatial and chemical control of crystal nucleation, regulation of crystal growth, crystal morphology and shell microstructure [9]–[10]. The outer calcite prismatic layer is always related to the proteins secreted from the outer epithelia cells of the edge of the mantle, whereas the inner aragonite nacreous layer is related to the proteins from the pallial region of the mantle [11]–[12].

Recent studies imply that the matrix proteins in the nacreous layer hold the key for the formation of aragonite crystals [13]–[15]. A number of proteins have been identified from *Pinctada fucata* shells and their functions in regulating the nacre biogenesis have been investigated [16]–[24]. Nacrein and MSI7 are both expressed in the pallial and edge regions of the mantle, which implies that they might take part in the formation of both the nacreous layer and prismatic layer of the shell [16]–[17]. Nacrein is the first molluscan shell matrix protein for which the entire sequence has been reported. It has a domain similar to carbonic anhydrase and obvious enzymatic carbonic anhydrase activity, which has been used as a biomineralization marker during shell formation [18]–[19]. MSI7 participates in forming the framework of the whole shell [17]. ACCBP, a novel extrapallial fluid protein that induces the formation of amorphous CaCO_3_ (ACC), was first separated by an ACC-binding method [20]. Pif80 [21], N19 [22], and N16 [23] are localized in the nacreous layer and involved in the formation of the aragonite crystals. MSI60 is the framework protein of the nacreous layer and plays a regulatory role in the nacre biomineralization [24]. The functions of these shell matrix proteins have mainly been investigated using *in vitro* crystallization experiments and by identifying their structures and patterns of expression. However, detailed functions remain to be determined *in vivo*, due to lack of an effective experimental system. The ‘flat pearl’ technique, first used by the U.C.Santa Barbara group [25], was utilized to observe the various nacre formations following steady-state growth interruption, and the growth and structure relationship in the initial stages of the shell biomineralization has been studied in detail [26]–[30]. In contrast, little is known about how the differential expression of the matrix proteins involved in inducing phase changes during crystal nucleation and growth control the phase of the deposited mineral. Although our previous study on pearl formation in *Pinctada fucata* suggested the role of matrix proteins in controlling the nacre growth [31], the study was hindered by the relatively slow growth of the pearls and the anatomical complexity of the capsular ‘pearl sac’ containing the secretory cells. Fortunately, flat pearl has proven to be one of the most attractive models for elucidating the process of molluscan shell formation. The one-to-one relationship between the expression of matrix proteins and the crystal polymorph *in vivo* might be clearly exhibited in this model.

This study aims to investigate the process of nacre biomineralization with periodic growth interruption during the early stages of nacre formation, including the gene expression patterns of matrix proteins. Scanning electron microscopy (SEM), Raman and FTIR spectroscopy were utilized to identify the nacre microstructure and the crystal phase constituting the flat pearl. Real-time PCR was performed at different stages for transcriptional profiles of genes encoding Nacrein, MSI7, N16, MSI60, Pif80, N19 and ACCBP. Our results estimate the roles of matrix proteins involved in flat pearl biomineralization *in vivo*, which is spatially and temporally regulated in a developmental sequence largely parallel to that at the growth front of the natural shell.

## Materials and Methods

### Animals

Pearl oysters, *Pinctada fucata* (with shells of 5.5–6.5 cm in length and 45–55 g of wet weight and about 2 years of age) were obtained from the Guofa Pearl Farm (Beihai, Guangxi Province, China). In our laboratory, the oysters were maintained at 20°C in an aquarium that contained aerated artificial seawater at 3% salinity.

### The inner-shell film preparation

The inner-shell film adheres to the inner surface of the shell so firmly that it cannot be removed unless treated with ethylenediaminetetraacetic acid (EDTA). The film collection was performed as described in Yan *et al* [32], with some modifications. The shells of *Pinctada fucata* were cut to proper sections with nacre. After being rinsed with EDTA (0.5 M) for 12 h, the film was separated from the section. The detached film was rinsed again with EDTA (0.5 M) until it was completely decalcified. The decalcified film was washed extensively with distilled water.

### Implantation procedure and sample collection

The implantation procedure was conducted as described by the U.C.Santa Barbara group [25]–[26], [33]–[34], with some modifications. Square microscope cover glass (approximately 36 mm^2^ by 0.15 mm thick) covered by inner-shell film and the cover glass used as a control were inserted into the extrapallial cavity (the region between the mantle and shell) of a total of 100 individuals. Care was taken to avoid damage to the mantle tissue. After implantation, oysters were returned to the seawater tanks for 5, 10, 15, 20, or 25 days. Implants remained in contact with the shell-facing side of the mantle organ throughout the duration of their incubation. At each sampling time, 18 oysters were randomly divided into three groups and sacrificed, and the mantle facing sides of the cover glass were pooled and kept in liquid nitrogen for total RNA extraction. Mantle tissues without facing sides of the cover glass were collected as controls. These implants were excised from the shell with a scalpel and dried for examination.

### Crystal characterization

The crystals deposited on the surfaces of implants were investigated using SEM and Raman spectroscopy *in situ*. The implants were coated with 10-nm-thick gold and analyzed using an FEI Quanta 200 scanning electron microscope (FEI,The Netherlands). The crystals were positioned on the implant and placed under a microscope (50×) that can focus the laser beam on the sample while collecting the backscattered light. The Raman spectroscopy of the crystals were recorded at an excitation wavelength of 514 nm. The spectra were scanned three times for 20 s in the range of 100 to 1400 cm^−1^ using a Renishaw RM2000 spectrometer. For FTIR investigations, the crystals were collected from the cover glass, and KBr pellets were produced with a 1% sample and analyzed on a Perkin-Elmer model 1600 FTIR spectrometer. All the spectra were recorded at 4 cm^−1^ resolution with 64 scans with a strong Norton-Beer apodization.

### Molecular analyses

Total RNA was extracted from the mantle using TRIzol®RNA Isolation Reagents (Life Technologies), according to the manufacturer’s instructions. The quantity of RNA was assessed by measuring OD_260/280_ with an Utrospec 3000 UV-visible spectrophotometer (Amersham Biosciences). The integrity of RNA was determined by electrophoresis on a 1.2% formaldehyde-denatured agarose gel stained with ethidium bromide. Then, high-quality total RNA was reverse transcribed to cDNA using the PrimeScript®RT reagent Kit (Takara) following the manufacturer’s instructions. Real-time PCR was conducted with the Mx3000P™ RT-PCR system (Stratagene) using a SYBR®Premix Ex Taq™II kit (Takara). The cycle conditions were as follows: 95°C for 30 s (1 cycle); 95°C for 5 s; 60°C for 20 s (40 cycles). Dissociation curves were analyzed to determine the purity of the product and specificity of the amplification. Seven genes encoding shell matrix proteins (Nacrein, N19, N16, Pif80, MSI60, MSI7 and ACCBP) were targeted. The housekeeping gene actin was selected as a reference for the calculation of relative expression levels of the genes. Primer sequences of each gene were outlined in Table S1.

### Statistical analysis

The cDNA of the three groups was detected at sampling time, and repeated three times for each sample. After data analysis, we standardized the 2^−ΔΔCt^ of each gene by taking the 5th day as the zero point. Data were compared by one-way analysis of variance (ANOVA) and with post hoc comparisons by Duncan's Multiple Range Test using the software SPSS 20.0. All tests were performed at a significance level of 5%.

## Results

### Microstructure of growth surfaces in the flat pearl

**Figure 1:**
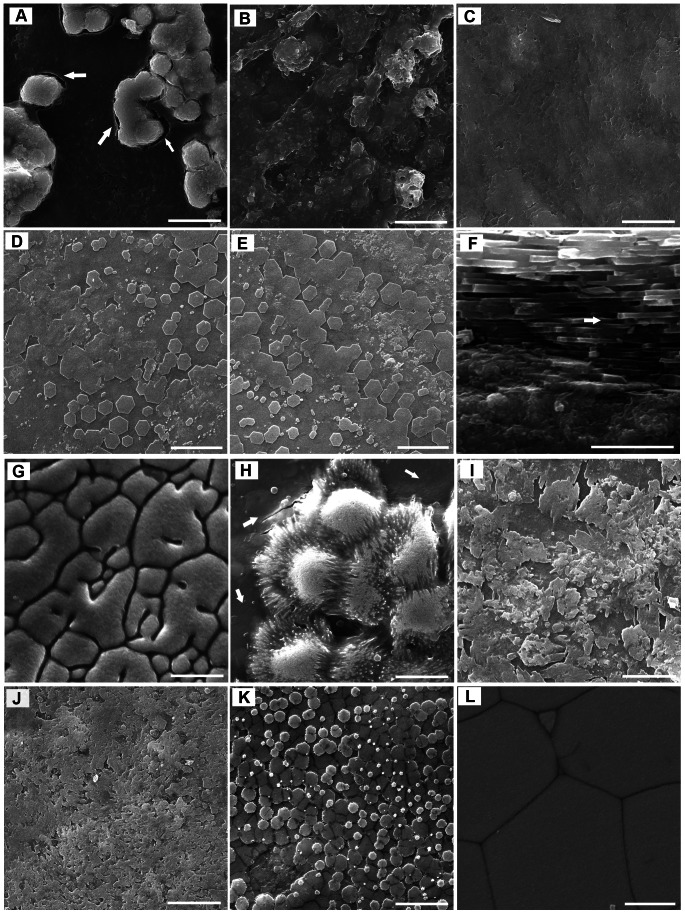
Scanning electron microscope images of the deposition on the surface. (A) 5d (the inner-shell film indicated by arrows), (B) 10d, (C) 15d, (D) 20d, (E) 25d and (F) cross section of 25d (brick and mortar structure indicated by arrows) in the inner-shell film system. Growth direction is towards the top in (F). (G) 5d, (H) 10d (the new forming organic film indicated by arrows), (I) 15d, (J) 20d and (K) 25d in the film-free system. (L) prismatic layer of adult shell. Scale bars, 10 µm in (A), (B), (C), (D), (E), (G), (H), (I), (J), (K) and (L), 4 µm in (F).

To investigate the process of nacre growth in the flat pearl, *in vivo* implantation experiments were conducted. The transitory morphologies leading to steady-state crystal growth after nacre growth interruption were observed. The sequential morphologies of this growth sequence are presented in Figure 1.

In the inner-shell film system, the crystals nucleated randomly on the implanted inner-shell film and they coalesced together to form the initial layer after 5 days of growth (Figure 1A), at which point the inner-shell film around the crystals can be observed (Figure 1A arrow). On the 10th day after implantation, the crystals had spread across the entire substrate, and the deposited layer had coarse surface (Figure 1B). Figure 1C shows that at 15 days, the morphology of deposited minerals became smoother than it had been at 10 days. The crystals packed into a compact aggregation and the surface appeared flattened. After 20 days, the hexagonal flat tablets appeared on the surface (Figure 1D), but some differently-shaped, bigger tablets were formed by the fusion of crystals with disordered patterns. After 25 days the surface showed a normal stair-like growth pattern, and the deposited crystals changed into the typical hexagonal flat tablets (Figure 1E) that are structurally similar to the nacre of *Pinctada fucata*. In addition, a brick and mortar arrangement was also observed in the cross section of the crystals at 25 days (Figure 1F arrow).

In the film-free system, compared with the inner-shell film system, the surface of the cover glass was completely covered by prism-like crystals at 5 days (Figure 1G). This prismatic structure is different from the calcitic prisms present in the outer shell of adult *Pinctada fucata* (Figure 1L). After 10 days, the spherical particles with acicular margins nucleated randomly on the surface, similar to the process at 5 days in the inner-shell film system (Figure 1H). The newly forming organic layer around the spherical particles was also visible (Figure 1H arrow). After 15 days, the surface was completely covered by deposited minerals which were composed of irregularly shaped crystals (Figure 1I). The crystals became smaller and packed into a compact aggregation, which resulted in a smooth surface at 20 days (Figure 1J). After 25 days, the crystals were amalgamated to form round tablets (Figure 1K), and the surface did not appear to have a normal stair-like growth pattern.

### Polymorph of deposited crystals on the surface

**Figure 2:**
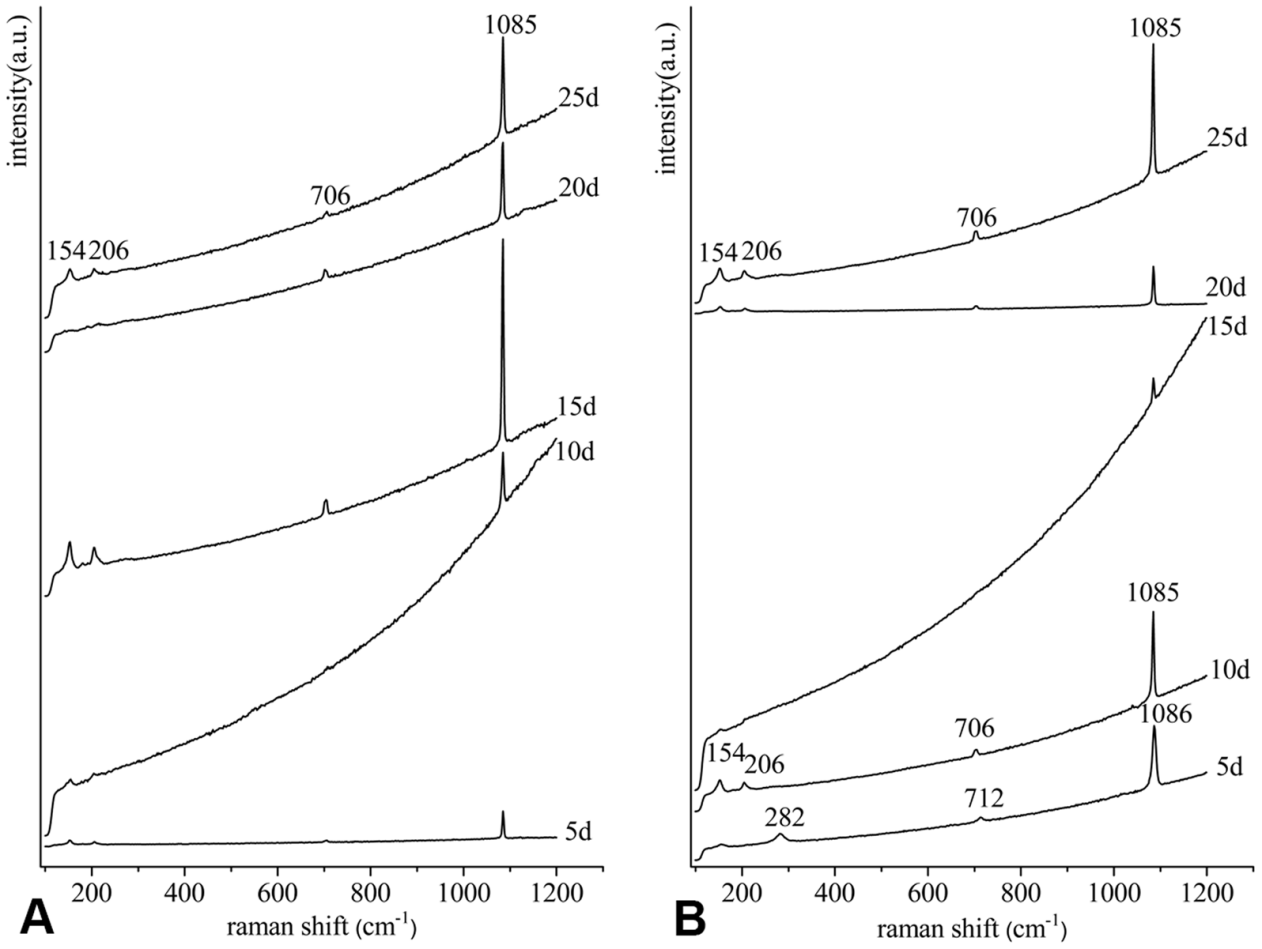
Raman spectra of the crystals deposited on the surface. (A) in the inner-shell film system, (B) in the film-free system. The characteristic peaks of calcite are at 282, 712 and 1086 cm^−1^ (5d in B), and those of aragonite are at 154, 206, 706 and 1085 cm^−1^.

To ascertain the polymorph of crystals deposited on the surface of the substrate, the same stages were analyzed by Raman and FTIR spectroscopy. The Raman spectra obtained from the periods of implantation are presented in Figure 2. In the inner-shell film system, all of the crystals from the growth periods were confirmed to be aragonite (Figure 2A), which exhibited characteristic aragonite bands at 154, 206, 706 and 1085 cm^−1^. The baseline of Raman spectra increased significantly from 5 to 10 days, decreased at day 15, and then showed a relatively steady level between days 15 and 25 (Figure 2A). In the film-free system, Raman analysis confirmed the crystals as aragonite (Figure 2B) except for the deposition on day 5. The Raman spectra revealed the crystals on the cover glass as calcite, with three bands at 282, 712 and 1086 cm^−1^ (Figure 2B). This is different from the polymorph of calcium carbonate in the inner-shell film system at 5 days. The baseline of Raman spectra also showed a different pattern from that in the inner-shell film system (Figure 2B). The baseline increased gradually from days 5 to 15, decreased suddenly at day 20, and then increased again at day 25 (Figure 2B).

**Figure 3:**
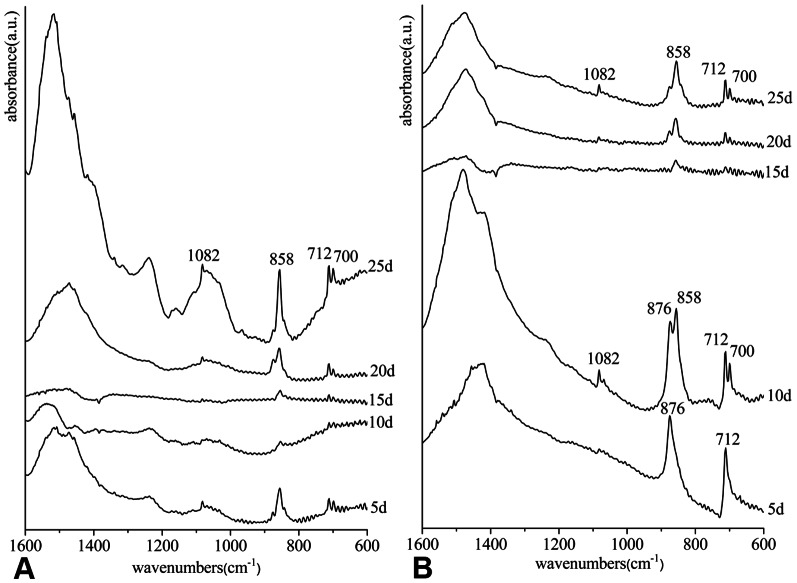
FTIR spectra of the crystals collected from the surface. (A) in the inner-shell film system, (B) in the film-free system. The characteristic bands of calcite are at 712 and 876 cm^−1^ (5d in B), and those of aragonite are at 700, 712, 858 and 1082 cm^−1^. The mixture of calcite and aragonite is seen at 10d in B.

Because Raman spectra can only identify small areas of crystals on the surface of the inner-shell film, it is inadequate to characterize the entire crystal. Therefore, we collected crystals grown on the inner-shell film and characterized them by FTIR in the inner-shell film system. The crystals on the surface of the film at each period demonstrated the typical bands of aragonite at 700, 712, 858 and 1082 cm^−1^ (Figure 3A), indicating that the crystals deposited in this system were all aragonite. In the film-free system, the spectrum of the crystals on the surface of the cover glass at day 5 contained the typical bands of calcite at 712 and 876 cm^−1^ (Figure 3B). The crystals over the other periods were confirmed to be aragonite by FTIR analysis. The characteristic bands of calcite and aragonite were observed to occur simultaneously at day 10 because of the mixture of calcite at day 5 and aragonite at day 10, and the characteristic bands of calcite disappeared with increasing aragonite content after day 15 (Figure 3B).

### Expression patterns of genes during the initial stages of nacre growth

**Figure 4:**
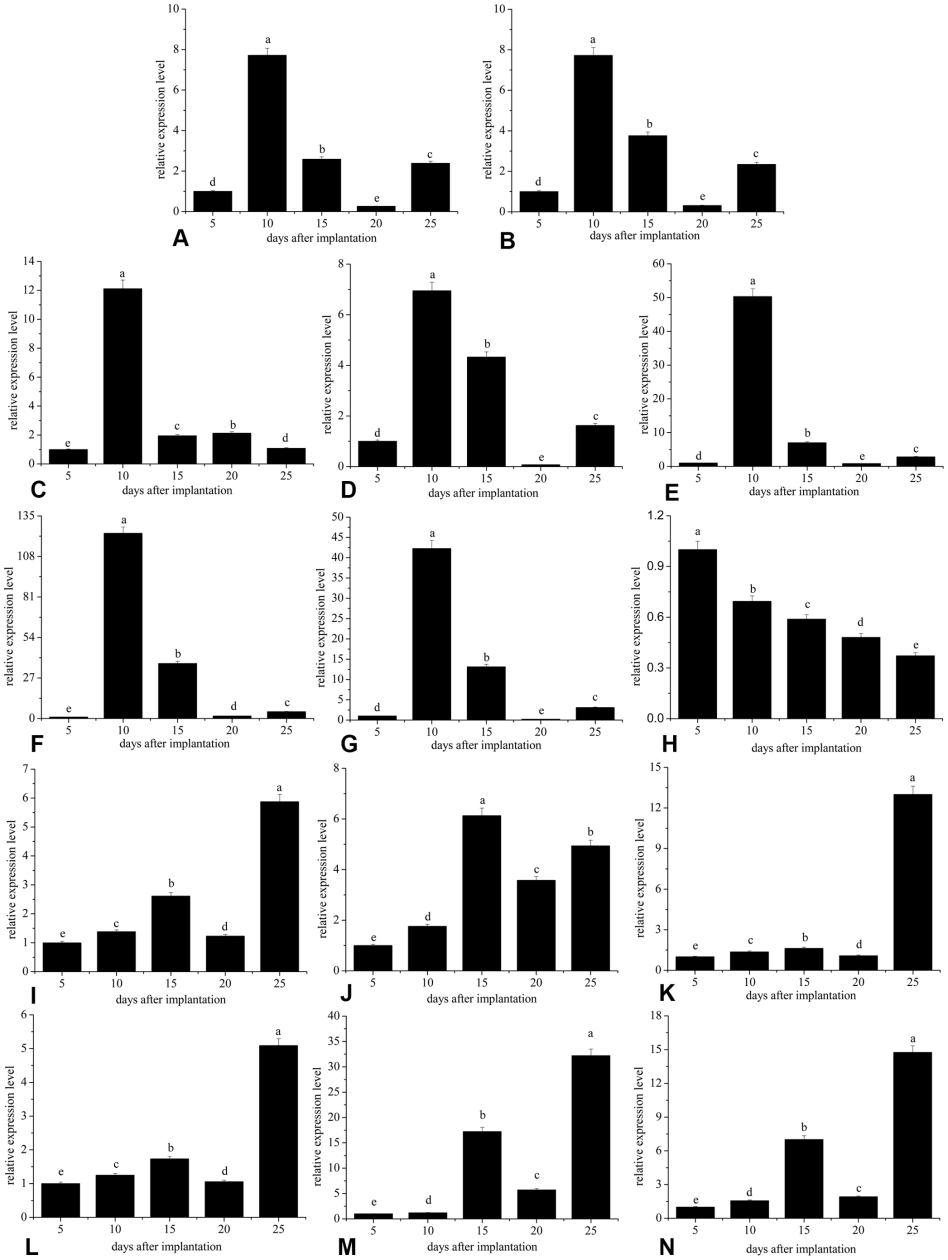
The relative expression of genes encoding matrix proteins at different stages of nacre growth. (A), (B), (C), (D), (E), (F) and (G) The relative expression levels of MSI7, Nacrein, ACCBP, MSI60, N19, N16 and Pif80 in the inner-shell film system, respectively. (H), (I), (J), (K),(L), (M) and (N) The relative expression levels of MSI7, Nacrein, ACCBP, MSI60, N19, N16 and Pif80 in the film-free system, respectively. Values in the same figure with a different superscript are significantly different (p<0.05).

In the inner-shell film system, the expression levels of MSI7, Nacrein, MSI60, N19, N16 and Pif80 showed similar patterns (Figure 4A,B,D,E,F,G), which increased significantly and reached a maximum at 10 days, and decreased suddenly at 15 days to their minimum value at 20 days, then increased at 25 days again. The expression level of ACCBP increased significantly and reached a maximum at 10 days, then decreased suddenly at 15 days and remained at low levels from 15 to 25 days (Figure 4C). In the film-free system, the expression level of MSI7 decreased gradually to a minimum value at day 25 (Figure 4H). The expression patterns of Nacrein, MSI60, N19, N16 and Pif80 were similar and increased gradually from 5 to 15 days, then decreased sharply at 20 days to their maximum value at 25 days (Figure 4I,K,L,M,N). The expression of ACCBP increased gradually from day 5 to day 15, and remained high from 15 to 25 days (Figure 4J).

**Figure 5:**
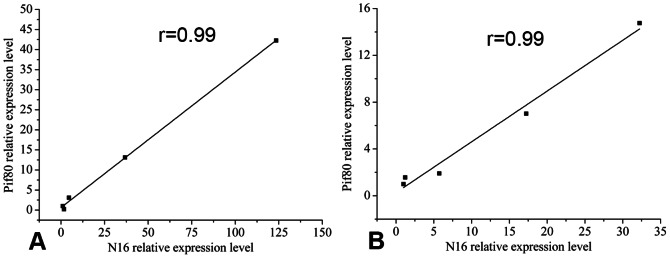
Correlation of gene expression levels between N16 and Pif80. (A) in the inner-shell film system, (B) in the film-free system. A significant correlation was observed between N16 and Pif80. r, correlation coefficient (N = 5).

As shown in Figure 5, strong correlations (r = 0.99) were observed between N16 and Pif80 in both the inner-shell film and film-free system. High levels of correlation between N16 and Pif80 indicated that Pif80 regulated the nacre formation with N16 *in vivo*, a finding similar to the report that Pif80 can induce aragonite crystals formation with N16 *in vitro* [21]. We also tested the expression levels of matrix proteins in the mantle facing the shell rather than the substrate. The relative expression levels of matrix proteins did not decrease or increase significantly during the growth periods in the control (Figure S1), which indicates that the normal growth of the pearl oyster did not disrupt the expression of matrix proteins. We are confident that the changes of these matrix proteins are related to nacre formation on the surface of the substrate.

## Discussion

The growth of nacre in the flat pearl of *Haliotis rufescens* is well studied by many researchers [13], [25], [26],[33],[34]. They focused on the relationship between growth and structure using material science methods. However, little attention has been paid to the expression of the matrix proteins that control the initial formation of the nacre. Furthermore, the towered nacre grown in gastropod mollusks does not contain the hexagonal flat tablets with the stair-like growth pattern that is uniquely characteristic of the growing surface of the nacre produced by bivalves [29].

In the present study, the nacre growth in the flat pearl has been investigated with a special emphasis on the onset of biomineralization. We show that biomineralization forming an organized composite microstructure can be induced on a substrate implanted between the mantle and the shell. This system provides unique access to the sequence of events governing this biofabrication. The same aragonite polymorphs of deposited calcium carbonate are on the surface of film-free and inner-shell film implants except for the different polymorphs at day 5 (Figure 1). The aragonites nucleated randomly on surface of the film in the inner-shell film system (Figure 1A), whereas the calcite crystals nucleated and covered the entire surface of the cover glass in the film-free system (Figure 1G). A nucleating organic layer is first secreted over the calcitic surface at 10 days in the film-free system (Figure 1H arrow), as observed by Fritz *et al* [25], and then aragonite particles nucleate randomly on the surface of the organic layer in a process similar to that in the inner-film system at 5 days (Figure 1A). Our observations support the previous suggestion that nano-sized aragonite crystals nucleate inside the dimples on the surface of the organic matrix that covers the outer prismatic columns in pearl oyster, *Pinctada fucata* [35]. The calcite-to-aragonite transition was also observed in abalone flat pearl and the oyster pearl biomineralization [25], [36]. However, the role of the inner-shell film in aragonite formation has not been well-studied. We proved that the switch in phase from calcite to aragonite requires the deposition of a new nucleating organic sheet *in vivo*. This finding might be explained by the strong interaction between the growing crystal and the surface of the inner-shell film, which may directly participate in calcite-to-aragonite transition as nucleation surface and predefined mold. The formation mechanism of the composite nacre is still under debate including the problem of the polymorph switching between calcite prism and aragonite nacres [37]. One explanation is based on a hetero-epitaxial nucleation model of aragonite tablets on the organic chitin sheet proposed by Weiner *et al* [2]. In this study the aragonite crystals are formed on the surface of the inner-shell film at 5 days, and the inner-shell film is an assembly of β-chitin frameworks, silk-like protein and acidic macromolecules [27]. These results imply that nucleation of aragonite crystals also can be explained by organic template or hetero-epitaxy at planar organic-inorganic interface.

At 25 days, the lamellar nacreous layers formed on the surface of the substrate in both of the two systems, and small irregular to round flat tablets of approximately 2–4 µm appeared in the film-free system (Figure 1K). However, typical hexagonal flat tablets with stair-like patterns were present in the inner-film system (Figure 1E). The results suggested that biomineralization on the substrate can be spatially and temporally organized so that the patterning of the composite closely resembles the growth front of the natural shell [25]. The nacre lamellae first appeared on the surface at 25 days in the film-free system (Figure 1K), and 5 days earlier, at 20 days, in the inner-film system (Figure 1D). This result indicated that the transition from initial mineral nucleation to steady-state mineral growth was largely affected by the initial organic matrix substrate [38]. The baseline of Raman spectra may reflect the actual contents of organic matrices in the deposition [31]. The highest baseline at day 10 in the inner-shell film system also appeared 5 days earlier, at day 15, in the film-free system during the first 20 days (Figure 2), suggesting that the formation of nacre lamellae was controlled by matrix proteins.

We describe *in vivo* studies of the relationship between matrix proteins expression and the deposited calcium carbonate in the presence of an inner-shell film. In the inner-shell film system, MSI7 exhibited an expression pattern similar to those of the other matrix proteins that regulate nacre biogenesis (Figure 4A). In contrast, MSI7 expression decreased continually from day 5 to 25 in the film-free system (Figure 4H). The highest level of MSI7 was detected at day 5, indicating MSI7 was involved in the formation of calcite. These results are consistent with a previous study suggesting that MSI7 might take part in the formation of both the nacreous layer and prismatic layer of the shell [17]. This suggests a role for inner-shell film in controlling aragonite nucleation and growth with matrix proteins *in vivo*, which has been confirmed *in vitro* [23], [39]–[41]. Namely, the soluble matrix is required for the controlled nucleation and growth of aragonite crystals on the insoluble matrix.

ACCBP is an ACC-binding protein that induces the formation of ACC, which is the transient precursor phase before aragonite or calcite [20]. The expression of ACCBP held comparatively steady from day 15 to 25 in both the inner-film and film-free systems, which suggests that ACCBP was involved in the synthesis of aragonite (Figure 4C,J). Nacrein has been thought to be involved in biomineralization by its carbonic anhydrase domain catalyzing HCO_3_-formation and supplying HCO_3_ ions for aragonite crystals, and plays an important role as synthesizer and carrier of aragonite in flat pearl formation [16], [18], [42]. MSI60 acts as nucleation surfaces and predefined molds and has a significant influence on the orientation of growing aragonite crystals, which lead to the formation of aragonitic tablet in nacre growth [24]. N19 [22], N16 [23] and Pif80 [21] are localized in the nacreous layer and regulate the formation of the aragonite crystals, so the expression of these proteins is closely related to the formation of aragonite on a surface. The expression of Nacrein, MSI60, N19, N16 and Pif80 had similar patterns during the first 20 days, and reached a maximum at day 10 in the inner-shell film system (Figure 4B,D,E,F,G). There were also similar patterns in the expression of these matrix proteins in the film-free system (Figure 4I,K,L,M,N), but they reached a maximum at day 15 during the early 20 days, 5 days later than in the inner-shell film system. These changes are in line with the results of Raman spectra baseline for the first 20 days (Figure 2). The maximum level of matrix proteins during the first 20 days corresponded to the formation of aragonite crystals over the entire surface (Figure 1B,I). This is the key step for the subsequent appearance of nacre lamella. These results indicate that the biofabrication of the flat pearl involves the secretion of matrix proteins from the adjacent mantle epithelial cells. These proteins may be responsible for the appearance of the nacre lamellae 5 days earlier on the surface in the inner-film system compared to the film-free system. Not only one protein is sufficient to induce the formation of the nacreous layer. Like Pif in shell formation, N16 was needed to achieve the full function of Pif97 and Pif80 in shell formation [21]. Similar expression patterns of Nacrein, MSI60, N19, N16 and Pif80 seem advantageous or even necessary for those proteins to function. Matrix proteins regulating the nacreous layer formation have the simultaneous expression pattern over the periods in the film-free and inner-film system respectively, indicating that these proteins are expressed in a coordinated way to produce elaborate and highly functional structures [11].

The highest expressions of Nacrein, MSI60, N19, N16 and Pif80 were detected at day 25 (Figure 4I,K,L,M,N), indicating that these proteins were involved in the appearance of the round flat tablets in the film-free system, as observed during pearl formation of *Pinctada fucata* [31]. However, the relatively low levels of MSI7, Nacrein, MSI60, N19, N16, and Pif80 between 15 and 25 days (Figure 4A,B,D,E,F,G) are related to the nacre formation from smooth surfaces into typical hexagonal flat tablets with a normal stair-like growth pattern in the inner-shell film system (Figure 1C,D,E). The increase or decrease in gene expression may be linked to the calcification rate [43], so that even the low expression of those proteins contributed to the gradual formation of the nacre lamellae with stair-like patterns. It has been reported that both N16 and N19 could inhibit the *in vitro* crystallization of calcite and the growth inhibition of undesired aragonite crystal faces is an effective way to modify the morphology of CaCO_3_ crystals and orient nacre growth [22], [23], [37], [44], [45], while Pif80 can induce the nucleation of aragonite crystals and is directly involved in creating the layered arrangement of aragonite platelets in nacre [21]. Crystal nucleation and growth inhibition are both antagonistic mechanisms that are involved in nacre formation [46], [47]. We hypothesized that the expression of these matrix proteins in the film-free and inner-film system can inhibit the spontaneous crystallization of aragonite and slow down the rate of aragonite precipitation to form fine microstructures in the nacre growth of flat pearl. Our results indicate that the deposition of aragonite crystals was controlled by matrix proteins.

Exposure of the mantle to foreign substrate stimulates a cellular response that initially activates a similar sequence of protein secretion and aragonite biomineralization. Our results are inclined to support that mantle epithelial cells engaged in nacre growth by secreting matrix proteins in a cell-mineral interface other than the extrapallial fluid cavity, which also supports the idea that matrix proteins are regionally expressed [11]. This challenge the prevailing model of shell formation which holds that calcium carbonate and the organic matrix are secreted in extrapallial fluid and that assembly into shell layers is matrix-mediated. The change in the model is that the initiation and control of molluscan shell biomineralization is a cellular process [48]. We suppose that the biological fidelity of molluscan shell architecture is a consequence of a dynamic interaction at the cell-mineral interface, in which cell recognition of the surface governs a genetic switch controlling the shell formation [25], [39]. These studies should provide additional insights into the molecular mechanisms governing the nacre biosynthesis. Further studies on the cellular mechanism of nacre formation *in vitro* will contribute to the synthesis of nacre in artificial systems.

## Supporting Information

Figure S1 **The relative expression of matrix proteins in the mantle facing the shell at different stages.** (A), (B), (C), (D), (E), (F) and (G) The relative expression levels of Nacrein, ACCBP, MSI60, N19, N16, Pif80 and MSI7 respectively. Values in the same figure with a different superscript are significantly different (p<0.05).
(TIF)

Table S1 **Primers sequences of genes used in real-time PCR analysis.**
(DOCX)
