# Correction: Patterns of Expression in the Matrix Proteins Responsible for Nucleation and Growth of Aragonite Crystals in Flat Pearls of *Pinctada fucata*

**DOI:** 10.1371/annotation/235dc29f-c950-4da9-8e86-ca4bff836873

**Published:** 2013-09-20

**Authors:** Liang Xiang, Jingtan Su, Guilan Zheng, Jian Liang, Guiyou Zhang, Hongzhong Wang, Liping Xie, Rongqing Zhang

In the Author Contributions statement, first author (LX) was incorrectly omitted from the list of those authors that "Conceived and designed the experiments." 

