# Supplementary figures and images for: Patterns of Expression in the Matrix Proteins Responsible for Nucleation and Growth of Aragonite Crystals in Flat Pearls of Pinctada fucata
Source: PLoS One. 2013 Jun 12;8(6):e66564. doi: 10.1371/journal.pone.0066564 (PMC3680448; doi:10.1371/journal.pone.0066564)

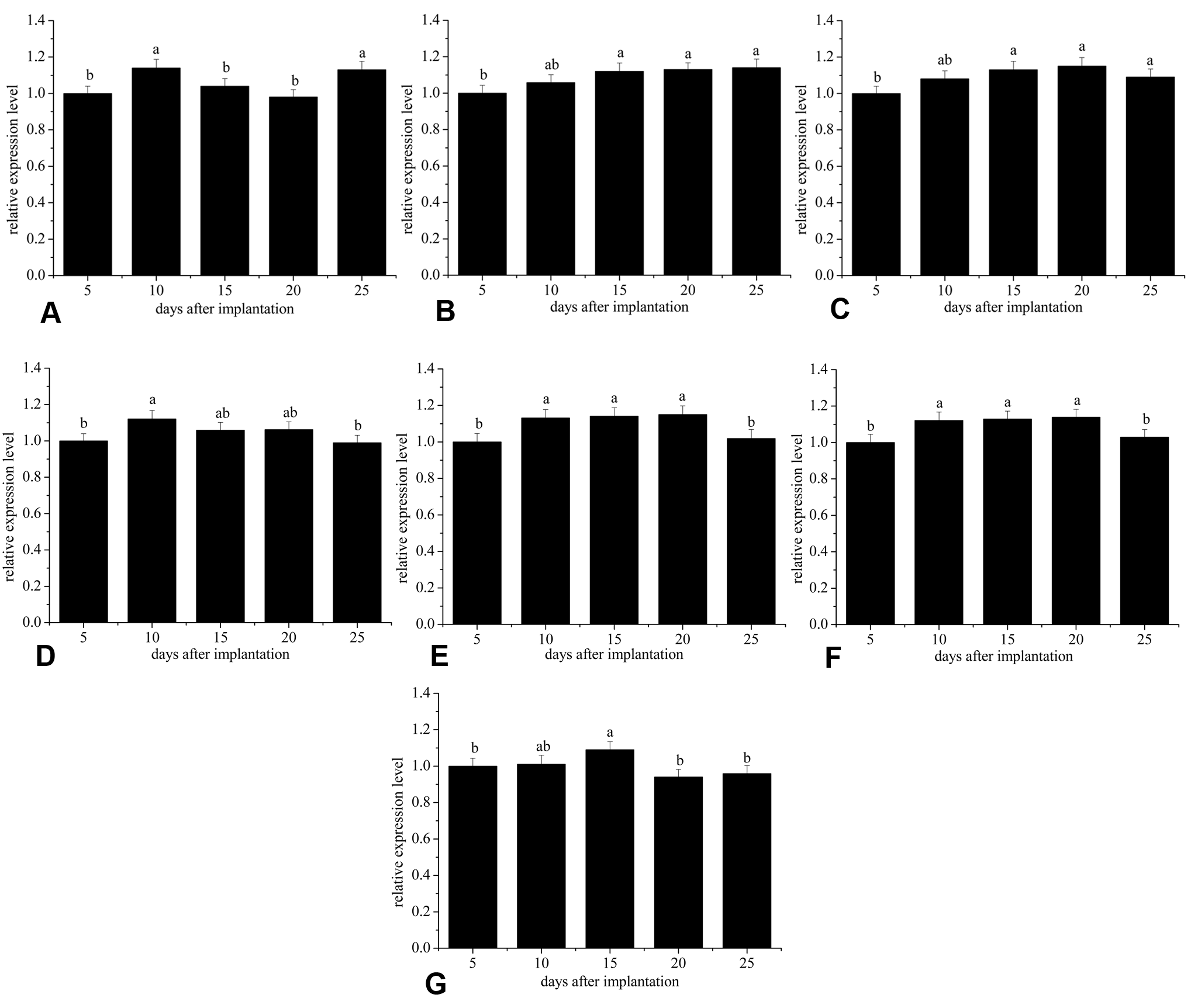

Supplement: Figure S1 — The relative expression of matrix proteins in the mantle facing the shell at different stages. (A), (B), (C), (D), (E), (F) and (G) The relative expression levels of Nacrein, ACCBP, MSI60, N19, N16, Pif80 and MSI7 respectively. Values in the same figure with a different superscript are significantly different (p<0.05). (TIF) [file pone.0066564.s001.tif]
