# Supplementary material for: Patterns of Expression in the Matrix Proteins Responsible for Nucleation and Growth of Aragonite Crystals in Flat Pearls of Pinctada fucata
Source: PLoS One. 2013 Jun 12;8(6):e66564. doi: 10.1371/journal.pone.0066564 (PMC3680448; doi:10.1371/journal.pone.0066564)
Supplement: Table S1 — Primers sequences of genes used in real-time PCR analysis. (DOCX) [file pone.0066564.s002.docx]

**Table S1. Primers sequences of genes used in real-time PCR analysis.**

| primer | sequence (5’-3’) |
| --- | --- |
| nacrein-F | GAGCCAGAGGATGGGGAAA |
| nacrein-R | GCCTCCATAGGTGTGAAACGA |
| N19-F | CCAGATTTCAACTCGATCTAAGGA |
| N19-R | CGCCATACCCATCAAAAGTG |
| N16-F | TGCGGACGTTACTCATACTGCT |
| N16-R | CCACATCTAAGCCACTCATACCTCT |
| Pif80-F | TGCTGCCATCACGTGAGTATG |
| Pif80-R | GACTTCCCTTTCTCACACTTCCA |
| MSI60-F | AGGTGGACGAAGAGGTAGAGGTAG |
| MSI60-R | AGCGGCAGCGGCAACATC |
| MSI7-F | GATCGTCTTAATTGCCTCTGTTTCT |
| MSI7-R | CTCCAAGCCCGATAACACCT |
| ACCBP-F | GACATGGAACAAAGATGGTGGA |
| ACCBP-R | CTGTGGCTGGAATGGTTGG |
| actin-F | CTCCTCACTGAAGCCCCCCTCA |
| actin-R | ATGGCTGGAATAGGGATTCTGG |
